# Oncological colorectal surgery during the COVID-19pandemic—a national survey

**DOI:** 10.1007/s00384-020-03697-6

**Published:** 2020-07-29

**Authors:** Maximilian Brunner, Christian Krautz, Stephan Kersting, Georg F. Weber, Benno Stinner, Stefan R. Benz, Robert Grützmann

**Affiliations:** 1 Department of surgery, University Hospital, Friedrich-Alexander-University, Krankenhausstraße 12, 91054 Erlangen, Germany; 2 Department of general surgery, Elbe Hospital, Bremervörder Straße 111, Stade, Germany; 3 Department of general surgery, Hospital Böblingen, Bunsenstraße 120, Böblingen, Germany

**Keywords:** Colorectal surgery, Oncological surgery, COVID-19 pandemic, SARS-CoV-2 infection, Surgical caseload

## Abstract

**Purpose:**

The aim of this study was to clarify the surgical supply situation of oncological colorectal patients in Germany during limitations of the OR caseload due to the COVID-19 pandemic.

**Methods:**

Between 11th and 19th April 2020, all members of a consortium of German colorectal cancer centers were invited to participate in a web-based survey on the current status of surgical care situation of colorectal cancer patients in Germany.

**Results:**

A total of 112 colorectal surgeons of 101 German hospitals participated in the survey. Eighty-seven percent of the participating hospitals had to reduce their total surgical caseload and 34% their surgical volume for oncological colorectal patients during COVID-19 pandemic. Restrictions of the surgical caseload were independent of the size of the hospital and the number of cases of COVID-19 in the federal state of the hospital. Sixteen percent of colorectal surgeons consider surgical limitations to be not justified and 78% to be justified only if the care of oncological patients is ensured. Ninety-five percent of the colorectal surgeons interviewed stated that all oncological colorectal patients with an indication for surgery should be operated in time, despite the current reservations for COVID-19 patients. For the majority of the respondents (63% and 51%, respectively), an extended waiting time for surgery of up to 2 weeks was acceptable for non-metastatic and metastatic patients, respectively.

**Conclusion:**

In Germany, there is a temporarily relevant reduction of surgical volume in oncological colorectal patients. Most colorectal surgeons stated that oncological colorectal surgery should not be compromised despite the measures taken during the COVID-19 pandemic.

## Introduction

The COVID-19 pandemic has led to relevant changes in global health care systems, particularly in surgical disciplines [1, 2]. In order to expand and maintain intensive care capacities for patients with SARS-CoV-2 infection, surgical caseload in German hospitals has been drastically reduced by German health authorities. This strategy aimed to mobilize staff for the intensive care units and to reduce the demand for post-operative intensive care unit beds. Although primarily elective non-oncological surgeries were no longer performed, oncological patients are increasingly affected by caseload restrictions in the OR [3]. Delays in the therapy of patients with colorectal cancer can lead to a deterioration of survival chances of these patients [4]. The effects of the current measures on oncological colorectal surgery in Germany and worldwide are not yet known. The aim of this national survey was therefore to investigate the current situation of surgical care of colorectal cancer patients and the opinion of German colorectal cancer surgeons on possible measures and therapy adjustments for colorectal cancer patients during the COVID-19 pandemic.

## Methods

On April 11th 2020, an online survey using Google Forms (Google LLC, Menlo Park California, USA) was sent by e-mail to all members of a consortium of German colorectal cancer centers (Arbeitsgemeinschaft der Deutschen Darmkrebszentren (ADDZ)). A reminder e-mail was sent to all members on April 16th, 2020. Participation was possible from April 11th, 2020 to April 19th, 2020.

For the subgroup analysis, participating hospitals were divided into federal states with a low and high frequency of COVID-19 cases. As a threshold value, 150 cases per 100,000 inhabitants were selected. The data on the number of COVID-19 cases per 100,000 inhabitants were taken from the Robert Koch Institute as of April 23rd, 2020. Federal states with over 150 cases per 100,000 inhabitants were Bavaria, Baden Württemberg, Hamburg, Saarland, and North Rhine-Westphalia. Tertiary referral hospitals were compared to all other hospitals. Additionally, hospitals were stratified according to their caseload per year using the median as threshold.

Interrater consensus was classified into three levels (high > 80%, moderate 60–80%, and low < 60% agreement among respondents).

Data analysis was performed with SPSS software (version 24.0). Metric and ordinal data were compared using the Student’s *t* test or Mann-Whitney *U* test. For categorical data, the Chi-square test was used. The threshold for statistical significance was *p* < 0.05.

## Results

**Fig. 1:**
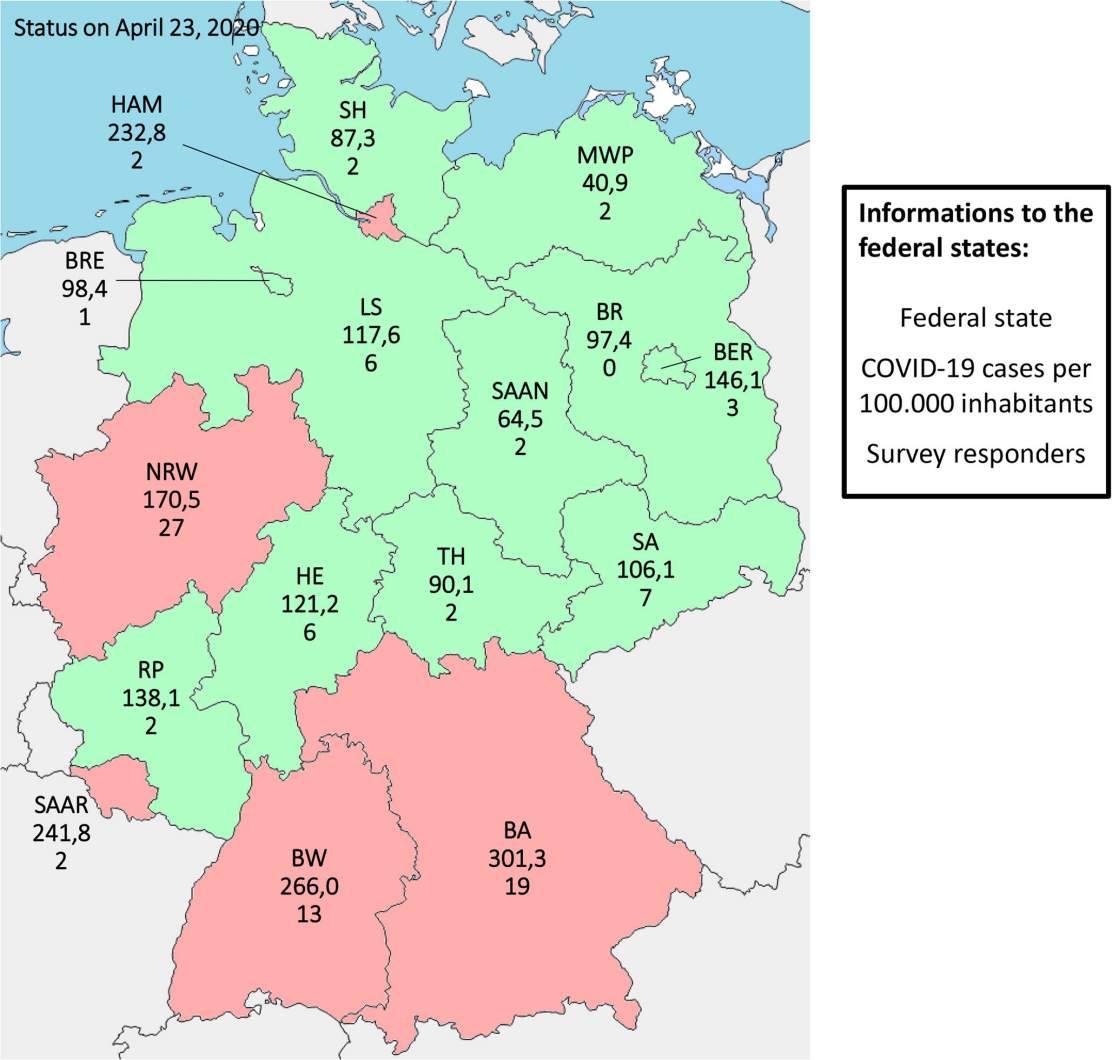
Distribution of participating hospitals to the different federal states in Germany and frequency of patients with COVID-19 in the different federal states (status in April 23, 2020; red = high frequency of COVID-19 (≥ 150 cases per 100,000 residents, green = low frequency of COVID-19 (< 150 cases per 100,000 residents); BA = Bavaria, BER = Berlin, BR = Brandenburg, BRE = Bremen, BW=Baden-Württemberg, HA = Hamburg, HE = Hesse, LS = Lower Saxony, NRW=North Rhine-Westphalia, RP = Rhineland Palatinate, SA = Saxony, SAA = Saarland, SAAN=Saxony-Anhalt, SH=Schleswig Holstein, TH = Thuringia, MWP = Mecklenburg Western Pomerania

A total of 112 colorectal surgeons from 101 German hospitals took part in this survey—including 42 tertiary referral hospitals (24 university hospitals, 18 non-university hospitals) and 59 other hospitals (mostly with a colorectal focus (*n* = 48)). The response rate in federal states with a high number of COVID-19 cases was significantly higher than in states with a low number of COVID-19 cases (*p* = 0.047) (Fig. 1). The average number of colorectal surgery cases of the participating hospitals was 123 cases per year with a range of 4 to 350 cases per year (Table 1).

**Table 1:** Data of participating hospitals

		*N* (%)
Number		101
Kind of hospital	Tertiary referral hospitals	42 (42)
	University hospital	24 (24)
	Non-university hospitals	18 (18)
	Other hospitals	59 (58)
	Hospitals with focus on colorectal surgery	48 (48)
	Basic care hospitals	11 (11)
Hospitals stratified to their federal state	North Rhine-Westphalia (NRW)	27 (27)
	Bavaria (BA)	19 (19)
	Baden-Württemberg (BW)	13 (13)
	Saxony	7 (7)
	Others	30 (30)
	Unknown	5 (5)
Hospitals stratified to frequence of patients with COVID-19 in their federal state (status on 23th april 2020)	< 150 cases per 100,000 residents	33 (34)
	> 150 cases per 100,000 residents (NRW, BA, BW, HA, SAA)	63 (66)
Caseload per year	Cases of oncological colorectal surgery per year	Mean 123 [range 4–750] Median 110
Hospitals stratified to caseload per year	< 110 cases per year	50 (50)
	≥ 110 cases per year	51 (50)

*NRW* North Rhine-Westphalia, *BA* Bavaria, *BW* Baden-Württemberg, *HA* Hamburg, *SAA* Saarland

Eighty-seven percent of the participating hospitals had to reduce their total OR volume due to the COVID-19 pandemic. The remaining caseload corresponded to ≤ 20% of the pre-pandemic surgical capacity in 10% of the hospitals, 21–40% in 37%, 41–60% in 28%, 61–80% in 11%, and 81–99% in 2%. Regarding oncological colorectal surgery, 34% of the participating hospitals had a reduced surgical caseload—6% of the hospitals had a remaining surgical volume of ≤ 20%, 8% of 21–40%, 9% of 41–60%, 9% of 61–80%, and 2% of 81–99%. There was no difference between tertiary referral and other hospitals and hospitals in federal states with high and low numbers of COVID-19 patients, both for the total surgical caseload limitations and for the surgical volume restrictions for oncological colorectal surgery (Table 2). The majority (78%) of the participating colorectal surgeons stated that the limitations in surgical capacities are only justified as long as the care of oncological patients is ensured. For 16% of those questioned, the limitations of the surgical capacity are not justified (Table 3).

**Table 2:** Current status of oncological colorectal surgery in Germany during COVID-19 pandemic; sub-groups included tertiary referral hospitals (TRH) vs. other hospitals (OH), hospitals with low caseload (< 110 cases per year, low-CL) vs. high caseload (> 110 cases per year, high-CL), hospitals in areas with low frequency of COVID-19 (< 150 cases per 100,000 residents, low-FRE) vs. with high frequency of COVID-19 (≥ 150 cases per 100,000 residents, high-FRE)

		All hospitals (*N* = 101)	Differences in sub-groups^a^
Remaining total surgical caseload	< 20%	10 (10)	–
	20–40%	37 (37)	
	40–60%	28 (28)	
	60–80%	11 (11)	
	80–99%	2 (2)	
	100%	13 (13)	
Remaining volume for oncological colorectal surgery	< 20%	6 (6)	–
	20–40%	8 (8)	
	40–60%	9 (9)	
	60–80%	9 (9)	
	80–99%	2 (2)	
	100%	67 (66)	
Changes in interdisciplinary tumor boards^a^	Unchanged	6 (6)	–
	Fewer staff	71 (70)	
	Fewer frequency	4 (4)	
	Via video conference	39 (39)	
Current procedure for COVID-19 patients	Surgery at the own hospital	95 (94)	
	Transfer to a specialized center	6 (6)	
Pre-surgery testing	All	20 (20)	OH vs. TRH: 12/59/29% vs. 31/36/33%, *p* = 0.008
	Patients with symptoms	50 (50)	
	None	31 (31)	
Used test method for COVID-19	PCR	97 (96)	–
	CT-Thorax	1 (1)	
	Clinical evaluation	3 (3)	
Lack of surgical equipment		8 (8)	–
Lack of protective equipment^b^		45 (45)	OH vs. TRH: 56% vs. 29%, *p* = 0.008

^a^Nineteen percent of the participating hospitals have more than one measure

^b^Masks, gowns, etc.

**Table 3:** Assessment of measures taken for the COVID-19 pandemic in Germany; sub-groups included tertiary referral hospitals (TRH) vs. other hospitals (OH), hospitals with low caseload (< 110 cases per year, low-CL) vs. high caseload (> 110 cases per year, high-CL), hospitals in areas with low frequency of COVID-19 (< 150 cases per 100,000 residents, low-FRE) vs. with high frequency of COVID-19 (≥ 150 cases per 100,000 residents, high-FRE)

		All hospitals (*N* = 101)	Differences in sub-groups*
Reduction of surgical caseload justified?	Yes, at all	6 (6)	–
	Yes, if care of oncological patients is ensured	79 (78)	
	No	16 (16)	
Measures taken for the COVID-19 pandemia regarding lifestyle appropriate?	Too much	16 (16)	–
	Adequate	81 (80)	
	Too little	4 (4)	
Measures taken for the COVID-19 pandemia regarding hospital management appropriate?	Too much	33 (33)	–
	Adequate	68 (67)	
	Too little	0 (0)	

Ninety-four percent of the participating hospitals operate on patients with SARS-CoV-2 infection at their hospital and 6% transfer those patients to specialized centers. A preoperative test for COVID-19 is always performed in 20% of the hospitals, while 50% of the hospitals test for symptoms and 31% do not perform preoperative COVID-19 tests. The preferred test method is the detection of SARS-CoV-2 by PCR; one clinic performs a thoracic CT-scan for COVID-19 detection (Table 2).

There are further consequences of the COVID-19 pandemic: 45% of the participating hospitals lack protective materials (masks, gowns), while hospitals with maximum care are significantly less affected (*p* = 0.008). A few hospitals (8%) suffer from a lack of surgical materials such as staplers. Interdisciplinary tumor boards are carried out in 70% of the hospitals with fewer staff and in 39% of the hospitals by video conference (Table 2).

Two thirds of all respondents stated that the measures taken for German hospitals during the COVID-19 pandemic are appropriate, but one third said they were too much. In comparison, measures in social life are considered as appropriate by 80% of respondents, 16% as too much, and 4% as too little (Table 3).

Assessment of statements and potential adjustments in therapy of oncological colorectal patients is shown in Table 4: 6 of 15 statements achieved a high, 3 a moderate, and 6 a low level of agreement.

**Table 4:** Assessments of 112 German colorectal surgeons on the therapy of oncological colorectal patients during COVID-19 pandemic. High/moderate/low agreement was defined as > 80%/60–80%/< 60% agreement. Sub-groups included tertiary referral hospitals (TRH) vs. other hospitals (OH), hospitals with low caseload (< 110 cases per year, low-CL) vs. high caseload (> 110 cases per year, high-CL), hospitals in areas with low frequency of COVID-19 (< 150 cases per 100,000 residents, low-FRE) vs. with high frequency of COVID-19 (≥ 150 cases per 100,000 residents, High-FRE), hospitals with low OR-caseload-reduction (remaining caseload > 40%, low-OCR) vs. high OP-caseload-reduction (remaining caseload ≤ 40%, high-OCR), hospitals with OP-caseload-reduction of colorectal patients (OCR-CR) vs. without OP-caseload-reduction of colorectal patients (non-OCR-CR)

	Agree	Indecisive	Disagree	Differences in sub-groups
High agreement				
(1) All patients with colorectal cancer and indication for surgery should undergo surgery in good time, regardless of the capacity reserve for COVID-19 patients	95%	3%	3%	–
What additional waiting time to surgery do you consider acceptable for patients with non-metastatic/metastatic colorectal carcinoma?				–
No additional waiting time	37%/49%			
Up to 2 weeks	63%/51%			
Up to 4 weeks	24%/16%			
Up to 8 weeks	3%/5%			
Up to 12 weeks	0%/2%			
(2) All patients with non-metastatic rectal cancer after neoadjuvant chemoradiation should receive a resection, regardless of the capacity available for COVID-19 patients	83%	8%	9%	–
(3) All patients with non-metastatic rectal cancer and indication for primary resection (T1–2 N0 M0) should currently receive neoadjuvant chemoradiation in order to maintain capacity for COVID-19 patients	4%	4%	92%	–
(4) All patients with metastatic colorectal carcinoma should currently preferably receive systemic treatment, even if a resection of the primary and/or metastases would make sense	3%	4%	94%	–
(5) Resectable colorectal liver metastases should currently preferably be treated using interventional procedures (e.g. ablation)	2%	6%	92%	–
(6) All oncological colorectal resections should currently be performed with appropriate protective clothing (FFP3-masks, safety glasses)	10%	9%	81%	–
Moderate agreement				
(1) In order to extend the time until the required operation, short-term radiation (5 × 5 Gray) should be avoided during the COVID-19 pandemic	20%	11%	70%	–
(2) The training of young colorectal surgeons should currently be put in the background to reduce morbidity and optimize the operating times	22%	15%	63%	–
(3) If the operating capacity is limited, patients with colorectal cancer should be selected based on their age and comorbidities	27%	11%	63%	–
Low agreement				
(1) In patients with rectal cancer and minimal residuals after neoadjuvant chemoradiation, a longer wait is justified during COVID-19 pandemic in order to possibly achieve a higher rate of complete remissions and thus less need for surgery	26%	15%	59%	–
(2) If the operating capacity is limited, all oncological patients should be selected based on their prognosis	38%	8%	55%	–
(3) Robotic oncological colorectal surgery should be avoided due to the longer surgical time to optimize surgical capacity during the COVID-19 pandemic	30%	19%	52%	Low-CL vs. high-CL: 27/29/44% vs. 32/9/60%, *p* = 0.022
(4) COVID-19-positive patients with colorectal cancer should be operated in specialized centers	43%	5%	52%	OH vs. TRH: 24/5/71% vs. 67/6/27%, *p* < 0.001 Low-FRE vs. high-FRE: 27/5/68% vs. 51/6/43%, *p* = 0.045
(5) All patients should receive a test for COVID-19 before planned surgery	38%	11%	51%	–
(6) Colorectal surgeons should currently be deployed in the therapy of COVID-19 patients in the absence of surgical capacity	36%	22%	43%	Low-FRE vs. high-FRE: 22/24/54% vs. 43/20/37%, *p* = 0.038

Highest agreement (95%) was achieved for the statement, that all oncological colorectal patients with an indication for surgery should not be postponed, despite the current OR volume reductions. For the majority of the respondents (63% and 51%, respectively), an extended waiting time for surgery of up to 2 weeks for non-metastatic, respectively, metastatic patients was acceptable, whereas 37% and 49%, respectively, did not consider additional waiting time appropriate for oncological colorectal patients.

There were three statements with low agreement that showed differences in the sub-group analysis: hospitals with a high number of colorectal surgery cases disagreed significantly more often with the statement that during the COVID-19 pandemic, the use of a surgical robot should be avoided to optimize OR capacity (60% vs. 44%, *p* = 0.022). Treatment of COVID-19-positive patients with colorectal cancer in specialized centers was advocated significantly more often by tertiary referral hospitals and hospitals in federal states with a high frequency of COVID-19 cases (67% vs. 24%, *p* < 0.001; 51% vs. 27%, *p* = 0.045). Colorectal experts from federal states with a high frequency of COVID-19 cases voted significantly more often for the use of colorectal surgeons in the intensive care of COVID-19 patients (43% vs. 22%, *p* = 0.038).

## Discussion

The COVID-19 pandemic has had a major impact on health care systems worldwide, leading to relevant caseload restrictions in general surgery, including colorectal cancer surgery. Hospitals were faced to a need of reorganization of their clinical practice. Important challenges included prioritization of surgical interventions, establishment of SARS-CoV-2 and non-SARS-CoV-2 emergency rooms, establishment of a SARS-CoV-2 surgical non-intensive care ward, establishment of a surgical SARS-CoV-2 operating area, and necessary precautions when using certain surgical techniques [2].

There are few reports from individual hospitals, particular from Italy, on strategies for colorectal surgery practice during the COVID-19 pandemic [3, 5–8]. It remains unclear how the COVID-19 pandemic affects the care of patients with colorectal cancer. In this regard, surveys may help to improve the limited evidence by providing a snapshot of current clinical practice and subsequently supporting clinical decision-making [9].

This nationwide survey reveals that health policy measures to save resources by reducing the overall number of surgeries during the COVID-19 pandemic also affect oncological colorectal resections in 34% of the hospitals. This fact is considered critical by the majority of interviewed colorectal surgeons. Regarding the treatment of oncological colorectal patients during the COVID-19 pandemic, the majority of the German surgeons surveyed plead for timely surgery with a maximum extension of the waiting period of 2 weeks and for compliance with national and international guidelines. However, questions remain open for discussion, in particular whether colorectal surgery for patients with SARS-CoV-2 infection should be performed in specialized centers and to what extent colorectal surgeons should be recruited for the ICU therapy of COVID-19 patients.

Of course, the care of oncological colorectal patients can vary from country to country, especially since Germany seems to be less affected by the COVID-19 pandemic than other nations according to the data from the Johns Hopkins Institute. Nevertheless, in times of scarce resources, an ethical discourse should be held on the extent to which a potential deterioration in the prognosis of oncological patients is justified for the care of other diseases in a highly developed industrial country.

## Conclusion

In Germany, health policy measures to save resources during the COVID19 pandemic led to a temporarily relevant reduction of surgical volume that affects oncological colorectal patients. Most of the participating German colorectal surgeons recommended that oncological colorectal surgery should not be compromised despite the measures taken during the COVID-19 pandemic.

## Data Availability

The manuscript including tables and figure contain all data.

## References

[1] Grasselli G, Pesenti A, Cecconi M (2020) Critical care utilization for the COVID-19 outbreak in Lombardy, Italy: early experience and forecast during an emergency response. JAMA. 10.1001/jama.2020.4031 10.1001/jama.2020.4031 32167538

[2] Flemming S, Hankir M, Ernestus RI, Seyfried F, Germer CT, Meybohm P, Wurmb T, Vogel U, Wiegering A (2020). Surgery in times of COVID-19-recommendations for hospital and patient management. Langenbeck's Arch Surg.

[3] Pellino G, Spinelli A (2020). How COVID-19 outbreak is impacting colorectal cancer patients in Italy: a long shadow beyond infection. Dis Colon Rectum.

[4] Roder D, Karapetis CS, Olver I, Keefe D, Padbury R, Moore J, Joshi R, Wattchow D, Worthley DL, Miller CL, Holden C, Buckley E, Powell K, Buranyi-Trevarton D, Fusco K, Price T (2019). Time from diagnosis to treatment of colorectal cancer in a South Australian clinical registry cohort: how it varies and relates to survival. BMJ Open.

[5] Lisi G, Campanelli M, Spoletini D, Carlini M (2020) The possible impact of COVID-19 on colorectal surgery in Italy. Colorectal Dis 22(6):641–642. 10.1111/codi.15054 10.1111/codi.15054 32227609

[6] Di Saverio S, Pata F, Gallo G, Carrano F, Scorza A, Sileri P, Smart N, Spinelli A, Pellino G et al (2020) Coronavirus pandemic and colorectal surgery: practical advice based on the Italian experience. Colorectal Dis 22(6):625–634. 10.1111/codi.15056 10.1111/codi.15056 32233064

[7] De Felice F, Petrucciani N (2020) Treatment approach in locally advanced rectal cancer during coronavirus (COVID-19) pandemic: long course or short course? Colorectal Dis 22(6):642–643. 10.1111/codi.15058 10.1111/codi.15058 32237263

[8] Gachabayov M, Dong XD, Latifi R, Bergamaschi R (2020). Considerations on colorectal cancer care in a COVID-19 pandemic epicenter. Surg Technol Int.

[9] Oba A, Stoop TF, Löhr M et al (2020) Global Survey on Pancreatic Surgery During the COVID-19 Pandemic. Ann Surg 272(2):e87-e93. 10.1097/SLA.0000000000004006 10.1097/SLA.0000000000004006 PMC7268883 32675507

